# One Health Risk and Disease (OHRAD): a tool to prioritise the risks for epidemic-prone diseases from One Health perspective

**DOI:** 10.1186/s41256-024-00359-w

**Published:** 2024-06-11

**Authors:** Sandul Yasobant, Priya Bhavsar, K. Shruti Lekha, Shailee Patil, Timo Falkenberg, Walter Bruchhausen, Deepak Saxena

**Affiliations:** 1 Department of Public Health Sciences, Indian Institute of Public Health Gandhinagar (IIPHG), Gujarat, India; 2 Centre for One Health Education, Research & Development (COHERD), Indian Institute of Public Health Gandhinagar (IIPHG), Opp. Air Force Head Quarters, Nr. Lekawada, Gandhinagar, 382042 Gujarat India; 3 School of Epidemiology & Public Health, Datta Meghe Institute of Medical Sciences (DMIMS), Wardha, India; 4 Global Health, Institute for Hygiene & Public Health, University Hospital Bonn, Bonn, Germany; 5 Geo Health, Institute for Hygiene & Public Health, University Hospital Bonn, Bonn, Germany

**Keywords:** Risk prioritisation, Disease prioritisation, One Health, Epidemic-prone diseases, OHRAD prioritisation tool

## Abstract

**Background:**

The rise in epidemic-prone diseases daily poses a serious concern globally. Evidence suggests that many of these diseases are of animal origin and contribute to economic loss. Considering the limited time and other resources available for the animal and human health sectors, selecting the most urgent and significant risk factors and diseases is vital, even though all epidemic-prone diseases and associated risk factors should be addressed. The main aim of developing this tool is to provide a readily accessible instrument for prioritising risk factors and diseases that could lead to disease emergence, outbreak or epidemic.

**Methods:**

This tool uses a quantitative and semi-quantitative multi-criteria decision analysis (MCDA) method that involves five steps: Identifying risk factors and diseases, Weighting the criteria, Risk and disease scoring, Calculating risk impact and disease burden score, and Ranking risks and diseases. It is intended to be implemented through a co-creation workshop and involves individual and group activities. The last two steps are automated in the MS Excel score sheet.

**Results:**

This One Health Risk and Disease (OHRAD) prioritisation tool starts with an individual activity of identifying the risks and diseases from the more extensive list. This, then, leads to a group activity of weighing the criteria and providing scores for each risk and disease. Finally, the individual risk and disease scores with the rankings are generated in this tool.

**Conclusions:**

The outcome of this OHRAD prioritisation tool is that the top risks and diseases are prioritised for the particular context from One Health perspective. This prioritised list will help experts and officials decide which epidemic-prone diseases to focus on and for which to develop and design prevention and control measures.

**Supplementary Information:**

The online version contains supplementary material available at 10.1186/s41256-024-00359-w.

## Introduction

The constant threat of epidemic-prone infections is a crucial public health concern globally and in India [1, 2]. There is an increase in newly epidemic-prone zoonotic diseases, especially in regions with a high host species diversity, intense animal-human interaction, and lower latitudes [3, 4]. Globally, around 60% of these infections were reported to be zoonotic [5]. In low-income countries, recently emerged zoonoses are estimated to contribute 26% of the disability-adjusted life years (DALYs) lost to infectious diseases and 10% of total DALYs lost [5]. In a nation like India, where human and animal populations are substantial and contact between the two is intimate, the control and prevention of such zoonotic diseases continue to be of utmost importance [6]. Simultaneously, the dynamic interplay of various socio-cultural factors and human-animal interaction adds further complexity to controlling these diseases [1, 7].

A One Health strategy is being promoted to effectively prevent and manage such diseases through intersectoral collaboration [8, 9]. However, such collaborations are limited to outbreaks and emergencies for a shorter time and are not sustained [10]. Although all the epidemic-prone diseases and the related risk factors should be addressed, considering the time and limited resources available for both animal and human sectors, it is necessary to identify the most pressing and most impactful risk factors and diseases. Joint prioritisation of epidemic-prone diseases can benefit both sectors as efforts are made to conduct effective and efficient surveillance, develop laboratory capacity, target outbreak response, implement disease control strategies, and identify research activities. Such a joint prioritisation will also help stakeholders to establish One Health collaborations by highlighting the most important diseases and risk factors to work on locally [11].

The prioritisation process is the act of putting tasks, problems, etc., in order of importance so that one can deal with the most important ones first [12]. Some specific tools are available for prioritising zoonotic or infectious diseases [11, 13, 14] and prioritising risk factors or hazards [15–17]. However, the available tools for risk factors are not particularly designed for the One Health context and thus have certain limitations. In most of these tools, a scoring system was based on qualitative, semi-quantitative and quantitative multi-criteria decision analysis (MCDA) methods. No specific tool is available to prioritise diseases and the risk factors of epidemic-prone diseases.

The One Health Risk and Disease (OHRAD) prioritisation tool was explicitly designed for One Health with reference to the Indian context. The main aim of developing this tool is to provide a readily accessible instrument for prioritising the risk factors and diseases that could lead to disease emergence, outbreak or epidemic.

## Methods

### OHRAD prioritisation tool principles

This tool development was an iterative process that followed the principles of quantitative and semi-quantitative MCDA and the Analytical Hierarchical Process (AHP). MCDA supports the decision-making process when multiple criteria need to be considered together to rank or choose the available alternatives [18]. The MCDA process does not change the judgment, but identifies, collects, and structures the information required by those making judgements to support the purposeful process. The MCDA can also support decision-making in healthcare as it improves transparency and consistency in decision-making [19]. The AHP was used to weigh the criteria. This process is generally used for ranking alternatives or selecting the best [20]. The AHP is one of the widely used multi-criteria methods.

### OHRAD prioritisation tool steps

The OHRAD tool includes five main steps (Table 1). The first step is an individual activity, the second and third steps are based on a group activity, and the last two steps are automated steps that can be performed using the pre-fixed automated MS Excel sheet (Supplementary material S1). The group activity should be preceded by the moderators, who are familiar with the tool and have a technical background in One Health. Five moderators are proposed for the subsequent sub-group activities.

**Table 1:** OHRAD prioritisation tool steps & prioritisation activity

Steps	Risk Prioritisation	Disease Prioritisation	Type of Activity
**Step 1**	Identifying Risk Factors	Identifying Diseases	Individual
**Step 2**	Weighting the Criteria	Weighting the Criteria	Group
**Step 3**	Risk Scoring	Disease Scoring	Group
**Step 4**	Risk Impact Score	Disease Burden Score	Automated
**Step 5**	Ranking Risks	Ranking Diseases	Automated

The details of the five steps of the tool are described below.

### Risk prioritisation

#### Step 1: Identifying risk factors

The first step of the tool was to identify the eligible risk factors from an extensive list, which would be prioritised in the next steps of the process. The list was prepared based on informal discussion and evidence searches. At the end of this step, a mutually agreed number of risk factors were selected for further prioritisation. The risk factors for further prioritisation were selected by summing up votes for each item. The factors that received the highest votes were selected.

#### Step 2: Weighting the Criteria

In this step, the pre-decided likelihood criteria were weighted using the AHP. The detailed definitions of each criterion are described below.

Risk is generally defined as the probability of an outcome having a negative effect on people, systems or assets [21]. For operationalisation in this tool, the risk function depends on the combined effects of likelihood and impact. Three criteria were pre-identified for measuring likelihood after previous discussion with experts: scope of exposure, frequency of exposure, and mitigation strategy. Thus, the risk function comprises three likelihood criteria: (1) exposed percentage of the population, (2) frequency (how frequently the exposure happens), and (3) availability of mitigation strategies for those exposed elements and one impact criterion: the potential for a severe disease outbreak.

##### Likelihood criteria

If the likelihood is defined as the chance that a certain event will happen [22], it can be analysed for the factors that favour an outbreak by using three criteria in the following ways:

1. ***Scope of Exposure***: The exposure is measured by the percentage of the population exposed to a particular risk factor. At the same time, this measure captures the vulnerability of this population.
2. ***Frequency of Exposure***: The frequency is the number of times that the population is exposed to a particular risk factor.
3. ***Mitigation Strategy***: Any availability of a risk mitigation strategy is considered. Risk mitigation strategy, in general, is the process used to prevent and reduce threats or their effects [23]. This means that a mitigation strategy may reduce all constituents of risk, hazard, exposure and vulnerability.

##### Impact Criterion

- ***Potential for Outbreak***: This is defined as the potential of that particular risk factor to cause an outbreak of any human disease. An outbreak is an increase (often sudden) in the number of disease cases above what is normally expected in the population. The term 'outbreak' is often used for a more limited geographic area [24].

#### Step 3: Risk scoring

Each risk factor was given a score in this third step. Questions were formulated for easy scoring and more uniform and accurate responses among the various groups for each criterion. Each risk factor will be scored based on all four criteria.

The details of the questions are presented in Table 2.

**Table 2:** Questions for scoring the indicators of risk factors

Indicators	Question	Score
**Likelihood indicator (three criteria)**		
Scope of Exposure	What proportion of the population is exposed to the particular risk factor?	1. Less than 20% population 2. 20–50% Population 3. More than 50% population
Frequency of Exposure	What is the estimated frequency of the exposure?	1. Improbable 2. Occasional 3. Frequently
Mitigation strategy	Is any strategy available to mitigate the risk?	1. Availability of strategy (policy/norms/regulations/ similar) 2. Availability of strategy (guidelines/ communication material/similar) 3. No strategy
**Impact indicator (one criterion)**		
Potential for outbreak	What is the potentiality of an outbreak of diseases in humans due to the concerned risk factor?	1. Low 2. Medium 3. High

#### Step 4: Risk impact score

Firstly, the score of each criterion was multiplied by its weight. Secondly, the weighted scores of the three criteria were summed and multiplied by the score of the impact criterion to obtain the final risk impact score.

$$\varvec{R}\varvec{i}\varvec{s}\varvec{k}\; \varvec{I}\varvec{m}\varvec{p}\varvec{a}\varvec{c}\varvec{t}\; \varvec{S}\varvec{c}\varvec{o}\varvec{r}\varvec{e}\;=\left(\left(\varvec{S}\varvec{c}\varvec{o}\varvec{p}\varvec{e}\; \varvec{o}\varvec{f}\; \varvec{e}\varvec{x}\varvec{p}\varvec{o}\varvec{s}\varvec{u}\varvec{r}\varvec{e}\;\varvec{*}\;\varvec{S}\varvec{c}\varvec{o}\varvec{p}\varvec{e}\; \varvec{o}\varvec{f}\; \varvec{e}\varvec{x}\varvec{p}\varvec{o}\varvec{s}\varvec{u}\varvec{r}\varvec{e}\; \varvec{w}\varvec{e}\varvec{i}\varvec{g}\varvec{h}\varvec{t}\right)+\left(\varvec{F}\varvec{r}\varvec{e}\varvec{q}\varvec{u}\varvec{e}\varvec{n}\varvec{c}\varvec{y}\; \varvec{o}\varvec{f}\; \varvec{e}\varvec{x}\varvec{p}\varvec{o}\varvec{s}\varvec{u}\varvec{r}\varvec{e}\;\varvec{*}\;\varvec{F}\varvec{r}\varvec{e}\varvec{q}\varvec{u}\varvec{e}\varvec{n}\varvec{c}\varvec{y}\; \varvec{o}\varvec{f}\; \varvec{e}\varvec{x}\varvec{p}\varvec{o}\varvec{s}\varvec{u}\varvec{r}\varvec{e}\; \varvec{w}\varvec{e}\varvec{i}\varvec{g}\varvec{h}\varvec{t}\right)+\left(\varvec{M}\varvec{i}\varvec{t}\varvec{i}\varvec{g}\varvec{a}\varvec{t}\varvec{i}\varvec{o}\varvec{n}\; \varvec{s}\varvec{t}\varvec{r}\varvec{a}\varvec{t}\varvec{e}\varvec{g}\varvec{y}\;\varvec{*}\;\varvec{M}\varvec{i}\varvec{t}\varvec{i}\varvec{g}\varvec{a}\varvec{t}\varvec{i}\varvec{o}\varvec{n}\; \varvec{s}\varvec{t}\varvec{r}\varvec{a}\varvec{t}\varvec{e}\varvec{g}\varvec{y}\; \varvec{w}\varvec{e}\varvec{i}\varvec{g}\varvec{h}\varvec{t}\right)\right)\varvec{*}\left(\varvec{P}\varvec{o}\varvec{t}\varvec{e}\varvec{n}\varvec{t}\varvec{i}\varvec{a}\varvec{l}\; \varvec{f}\varvec{o}\varvec{r}\; \varvec{o}\varvec{u}\varvec{t}\varvec{b}\varvec{r}\varvec{e}\varvec{a}\varvec{k}\right)$$

#### Step 5: Ranking risks

Once the risk impact score was generated for all the selected risks, the weighted scores were normalised using the formula below.

$$\boldsymbol{Normalised score}\; \boldsymbol{=}\; \frac{\boldsymbol{Risk}\; \boldsymbol{impact} \;\boldsymbol{score}\; \boldsymbol{of}\; \boldsymbol{particular} \; \boldsymbol{risk} \; \boldsymbol{factor}\; \boldsymbol{-} \;\boldsymbol{minimum} \; \boldsymbol{value}}{\boldsymbol{maximum} \; \boldsymbol{value} \; \boldsymbol{-} \; \boldsymbol{minimum}\; \boldsymbol{value}}$$

Lastly, the ranking was generated automatically based on the normalised score of each risk factor. These normalised scores were ranked group-wise, and a combined rank was also calculated.

### Disease prioritisation

#### Step 1: Identifying diseases

The first step of the tool for the disease prioritisation process was to select the diseases to be prioritised from an extensive disease list. The list was prepared based on informal discussion and evidence searches. At the end of this first step, a mutually agreed number of diseases were selected.

#### Step 2: Weighting the Criteria

In this step, the pre-decided criteria for disease threats were weighted using the AHP. The detailed definitions of each criterion are described below.

Four criteria were identified: severity, prevalence, transmissibility, and prevention or control strategies. As in the general risk model, these integrate aspects of the hazards, in this case, the relative properties of the pathogen, the exposure, i.e. the chance to get in contact with the pathogen, and the vulnerability, i.e. the probability of the pathogen leading to an outbreak in the population. This accords to the triangle in the agents, environments and hosts paradigm. The prevention and control strategies may affect all three conditions: the dangers from the pathogen (hazards/agents), the contacts (exposure/environment) and proneness to outbreaks (vulnerability/hosts).

##### Criteria for disease threats

1. ***Severity***: Severity is captured as the case fatality rate (CFR). CFR is the proportion of persons with a particular condition (e.g., patients with a certain disease) who die from that condition [25].
2. ***Prevalence***: Prevalence is the number of people with a specific disease or condition in a given population at a specific time. This measure includes newly diagnosed and pre-existing cases of the disease [26] and serves as a proxy for the likelihood of contact or exposure to the pathogen in the environment.
3. ***Transmissibility***: Transmissibility is measured by the ability to cause an epidemic. Epidemic refers to an increase, often sudden, in the number of cases of a disease above what is normally expected in that population in that area [27]. Thus, it indicates the particular vulnerability of the host population to a pathogen.
4. ***Prevention and control strategy***: Disease prevention and control strategies include interventions like vaccination, diagnosis, therapeutics, isolation/quarantine, and availability of communication material.

##### Outcome Indicator 

- ***Disease burden***: The term burden of disease generally describes the total, cumulative consequences of a defined disease or a range of harmful diseases with respect to deaths and disability in a community. The consequences include health, social aspects, and costs to society [28]. In metric terms, the burden is usually captured as the DALYs and economic loss equivalent. Due to the lack of data on the disease-specific and local burden of disease, only the joint estimate of the participants is used in this assessment. Due to the special scope of One Health and zoonotic diseases, both human and animal health are considered.

#### Step 3: Disease scoring

Each of the selected diseases was given a score in this third step. For each pre-decided criterion (four criteria for disease threats and one outcome indicator), questions were formulated for easy scoring and more uniform and accurate responses among the various groups.

The details of the questions are presented in Table 3.

**Table 3:** Questions for scoring the indicators for diseases

Indicators	Question	Score
**Criteria for disease threats**		
Severity	What is the CFR in humans and animals?	1. CFR < 5% in one or both sectors 2. CFR in one or both sectors ≥ 5—< 15% 3. CFR one or both sectors ≥ 15%
Prevalence	What is the estimated prevalence of this disease?	1. < 1 per 100,000 population 2. 1–100 per 100,000 population 3. > 100 per 100,000 population
Transmissibility	Has this disease caused an epidemic (human or animal) in your region?	1. Never or more than 10 years ago 2. Within the past ≥ 5 and < 10 years 3. Within the past 5 years
Preventive and control strategy	Is a strategy for disease prevention and control readily available? (includes diagnostic capacities, therapeutics and vaccinations)	1. Available for animals and humans 2. Available for animals or humans 3. Not available for animals and humans
**Outcome indicator**		
Disease burden	Is the disease a burden among humans and animals?	1. Neither 2. High DALY among humans or High economic loss due to animal diseases or animal loss equivalent 3. High DALY among humans and High economic loss due to animal diseases or animal loss equivalent

#### Step 4: Disease Burden score

Firstly, the score of each criterion was multiplied by its weight. Secondly, the four weighted criteria scores were summed and then multiplied by the outcome indicator (disease burden) score to obtain the disease burden score.

$$\varvec{D}\varvec{i}\varvec{s}\varvec{e}\varvec{a}\varvec{s}\varvec{e}\; \varvec{B}\varvec{u}\varvec{r}\varvec{d}\varvec{e}\varvec{n}\; \varvec{S}\varvec{c}\varvec{o}\varvec{r}\varvec{e}\;=\left(\left(\varvec{S}\varvec{e}\varvec{v}\varvec{e}\varvec{r}\varvec{i}\varvec{t}\varvec{y}\; \varvec{*}\; \varvec{S}\varvec{e}\varvec{v}\varvec{e}\varvec{r}\varvec{i}\varvec{t}\varvec{y}\; \varvec{w}\varvec{e}\varvec{i}\varvec{g}\varvec{h}\varvec{t}\right)+\left(\varvec{P}\varvec{r}\varvec{e}\varvec{v}\varvec{e}\varvec{l}\varvec{a}\varvec{n}\varvec{c}\varvec{e}\;\varvec{*}\varvec{P}\varvec{r}\varvec{e}\varvec{v}\varvec{e}\varvec{l}\varvec{a}\varvec{n}\varvec{c}\varvec{e}\; \varvec{w}\varvec{e}\varvec{i}\varvec{g}\varvec{h}\varvec{t}\right)+\left(\varvec{T}\varvec{r}\varvec{a}\varvec{n}\varvec{s}\varvec{m}\varvec{i}\varvec{s}\varvec{s}\varvec{i}\varvec{b}\varvec{i}\varvec{l}\varvec{i}\varvec{t}\varvec{y}\; \varvec{*}\varvec{T}\varvec{r}\varvec{a}\varvec{n}\varvec{s}\varvec{m}\varvec{i}\varvec{s}\varvec{s}\varvec{i}\varvec{b}\varvec{i}\varvec{l}\varvec{i}\varvec{t}\varvec{y}\; \varvec{w}\varvec{e}\varvec{i}\varvec{g}\varvec{h}\varvec{t}\right)+\left(\varvec{P}\varvec{r}\varvec{e}\varvec{v}\varvec{e}\varvec{n}\varvec{t}\varvec{i}\varvec{v}\varvec{e}\; \varvec{a}\varvec{n}\varvec{d}\;\varvec{c}\varvec{o}\varvec{n}\varvec{t}\varvec{r}\varvec{o}\varvec{l}\;\varvec{S}\varvec{t}\varvec{r}\varvec{a}\varvec{t}\varvec{e}\varvec{g}\varvec{y}\; \varvec{*}\varvec{P}\varvec{r}\varvec{e}\varvec{v}\varvec{e}\varvec{n}\varvec{t}\varvec{i}\varvec{v}\varvec{e}\;\varvec{c}\varvec{o}\varvec{n}\varvec{t}\varvec{r}\varvec{o}\varvec{l}\; \varvec{s}\varvec{t}\varvec{r}\varvec{a}\varvec{t}\varvec{e}\varvec{g}\varvec{y}\; \varvec{w}\varvec{e}\varvec{i}\varvec{g}\varvec{h}\varvec{t}\right)\right)\varvec{*}\left(\varvec{D}\varvec{i}\varvec{s}\varvec{e}\varvec{a}\varvec{s}\varvec{e}\; \varvec{b}\varvec{u}\varvec{r}\varvec{d}\varvec{e}\varvec{n}\right)$$

#### Step 5: ranking the diseases

The weighted scores were normalised, once the disease burden score was generated for all the selected diseases.

$$\boldsymbol N\boldsymbol o\boldsymbol r\boldsymbol m\boldsymbol a\boldsymbol l\boldsymbol i\boldsymbol s\boldsymbol e\boldsymbol d\boldsymbol\;\boldsymbol s\boldsymbol c\boldsymbol o\boldsymbol r\boldsymbol e\;\boldsymbol=\boldsymbol\;\frac{\mathbf D\mathbf i\mathbf s\mathbf e\mathbf a\mathbf s\mathbf e\boldsymbol\;\mathbf b\mathbf u\mathbf r\mathbf d\mathbf e\mathbf n\boldsymbol\;\mathbf s\mathbf c\mathbf o\mathbf r\mathbf e\boldsymbol\;\mathbf o\mathbf f\boldsymbol\;\mathbf p\mathbf a\mathbf r\mathbf t\mathbf i\mathbf c\mathbf u\mathbf l\mathbf a\mathbf r\boldsymbol\;\mathbf d\mathbf i\mathbf s\mathbf e\mathbf a\mathbf s\mathbf e\boldsymbol\;\boldsymbol-\boldsymbol\;\mathbf m\mathbf i\mathbf n\mathbf i\mathbf m\mathbf u\mathbf m\boldsymbol\;\mathbf v\mathbf a\mathbf l\mathbf u\mathbf e}{\mathbf{\left({maximum\;value\;-\;minimum\;value}\right)}}$$

Lastly, these normalised scores were ranked group-wise, and a combined rank was calculated.

### OHRAD prioritisation tool implementation

This tool was designed to be implemented in a co-creation workshop. One prerequisite for using this tool is that an extensive list of risk factors and the list of epidemic-prone diseases should be readily available before the commencement of the workshop. This list should contain a maximum of 50 risk factors and 50 diseases. These risk factors and diseases should be relevant to the local context.

This tool should be administered at the national/regional/local level among the key stakeholders across the animal husbandry, forestry, wildlife, environment and climate change and health department and the other potential stakeholders (including One Health experts, Public Health experts, practitioners from the human and veterinary departments, academic institutes and research institutes and community-based organisations). A total of 20–30 participants can be accommodated in a workshop based on the local context.

## Results

The risks and diseases are prioritised separately; however, both include the same steps.

### Step 1: Identifying risk factors and diseases

In this first step, all stakeholders are provided with an extensive list of risk factors and diseases. Then, each participant should provide their consent on whether that particular risk factor or disease should be included in the prioritisation exercise or not. The participants individually choose the risk factor or disease based on their experience and perception for inclusion in the prioritisation exercise.

The link to the two Google forms is provided to all stakeholders. The first form includes the list of risk factors, while the other is the list of diseases. They have to respond to each risk factor or disease and determine whether it should be included for prioritisation or not. There is also a feasible option for including new risk factors or diseases. In the end, based on the consensus among the stakeholders, it will be decided whether it should be included for further prioritisation or not. The selection of the number of risk factors and diseases can vary depending on the context and types of stakeholders invited to be part of the workshop.

### Step 2: Weighting the criteria

Once the risks and diseases are selected for prioritisation in the first step, the criteria are weighted using the AHP in this step. Weighting criteria is a group activity. All participants should be divided into groups of five to six participants. Each group should have representations from the different stakeholder groups (animal husbandry, forestry, environment and climate change, wildlife and health). The number of participants in the group can vary based on the total number of participants present. However, the total group number should not exceed five, irrespective of the total number of participants.

In AHP, scores are given based on a pair-wise comparison of all criteria, which means each criterion is compared to other criteria, and scores are given based on which criterion is more important than the others. All possible combinations will be scored between 1 and 9 (1 = equal importance to 9 = extreme importance).

Each group individually scores the criteria for risk factors using a pair-wise comparison of three pre-decided likelihood criteria (i.e. 9 combinations). For diseases, each group individually scores the criteria using a pair-wise comparison of four pre-decided criteria for disease threats (i.e. 16 combinations).

### Step 3: Risk & Disease scoring

This is again a group activity, and the same groups from the previous step will continue in this step. The score is given based on the decided questions and scoring pattern for each pre-decided criterion. Each group participant shares their opinions for the scoring based on their knowledge and experience. Any discrepancies in views for the scoring should be resolved through consensus building within the group. In such cases, moderators play a vital role in consensus building and reaching a group conclusion.

Risk Scoring: Each risk factor is scored on three pre-decided likelihood criteria (exposure, frequency and mitigation strategy) and a single impact criterion (potential for an outbreak).

Disease Scoring: Each disease is scored on four pre-decided criteria for disease threats (severity, prevalence, transmissibility, counter-strategy) and a single impact criterion (disease burden).

### Step 4: Risk impact score & Disease burden score

There are no specific inputs needed from the stakeholders of the workshop for this step. Each risk’s impact score and each disease’s burden will be automatically calculated once the information from the previous steps is filled in.

### Step 5: Ranking risks & diseases

The ranking is also automatically generated based on the risk impact and disease burden scores.

In future implementations, there might be instances where the scores of risk factors/diseases are the same. In such cases, stakeholders who are the workshop participants can decide the ranks for the risk factors/diseases with the same scores based on consensus. This will be completely up to the stakeholders as this tool is designed based on decision-making principles.

## Discussion

The proposed OHRAD prioritisation tool offers a process through which risk factors and diseases can be prioritised to inform disease prevention and control measures. This tool uses quantitative and semi-quantitative methods to prioritise risk factors and epidemic-prone diseases, which is also used in other tools of disease prioritisation [11].

This tool was developed considering the Indian context and was found to be simple to be implemented and operationalised. One of its strengths is that risk factors and diseases can be prioritised using a single tool. Secondly, being simple, it is easy to administer at the national, regional or local level and can be easily interpreted. Although the criteria are pre-decided, they can be changed or adjusted according to the local context.

In the pilot trial of the tool, it was possible to complete the prioritisation of the risks and diseases in a single-day workshop. The moderators play an important role in facilitating the discussion and extracting the required data for steps 2 and 3. Simultaneously, the data gathered from all the groups are entered into the automated Excel sheet by one designated individual. Equal input from all stakeholders is received throughout the individual and group activities of the selection and prioritisation. The participants and their background significantly impact the weighting and scoring of the risks and diseases, which may cause some bias in the scoring. Therefore, the grouping of the participants was done prior to the workshop and divided in such a way that there should be an equal representation from all the different departments in each group. Each group consists of a maximum of 6–7 participants. Due to the automated calculations, it was possible to compile the data of all the groups and present summary findings at the end of the workshop.

This tool is designed to prioritise risk factors for epidemic-prone diseases in the Indian context. However, it has huge potential to be validated further not only for other diseases but also in diverse geographic contexts. Therefore, we urge to One Health experts to validate this OHRAD prioritisation tool in different geographic contexts.

## Conclusions

The OHRAD prioritisation tool was developed to be used at the national/ regional/ local level among key stakeholders, organisations, and academic institutions interested in prioritising risk factors and epidemic-prone diseases. The operationalisation of this tool may differ among the different users based on their needs; however, it ultimately helps identify the risk factors and epidemic-prone diseases that need attention. This tool involves group decision-making processes and can produce results despite limited data available on individual diseases and risk factors in a low-resource setting. The final outcome of the tool will help the stakeholders develop strategies for prioritising risks and diseases for emergence and potentially for outbreaks or epidemics.

### Supplementary Information

Additional file 1: S1. Automated MS Excel sheet of the OHRAD prioritisation tool.

## Data Availability

All the information is embedded in the manuscript, and an automated sheet is provided as supplementary material [See Supplementary material S1].

## Abbreviations

AHP: Analytical heirarchial process
CFR: Case fatality rate
DALYs: Disability-adjusted life years
MCDA: Multi-criteria decision analysis
OHRAD: One Health Risk and Disease
